# Systematic Intervention with Formal Caregivers to Promote Nutritional Health of Older People with Dementia: An Impact Evaluation Study

**DOI:** 10.3390/ijerph22060849

**Published:** 2025-05-29

**Authors:** Paola Sarmiento-González, María Elisa Moreno-Fergusson, Alejandra Rojas-Rivera, Juan Alcides Cuadros-Mojica, Bibiana Ramírez-Pulido, Beatriz Sánchez-Herrera

**Affiliations:** 1 School of Nursing and Rehabilitation, Universidad de La Sabana, Chia 250001, Colombia; paola.sarmiento1@unisabana.edu.co (P.S.-G.); mariae.moreno@unisabana.edu.co (M.E.M.-F.); 2 School of Nursing and Midwifery, Universidad de Los Andes, Santiago 12455, Chile; arojas@uandes.cl; 3 Puravida Fundación, Bogota 110111, Colombia; juancuadros@puravidafundacion.org; 4 EICEA, School of Gastronomy, Universidad de La Sabana, Chia 250001, Colombia; bibiana.ramirez@unisabana.edu.co

**Keywords:** caregivers, dementia, geriatric nursing, nutrition, nursing methodology research, health impact assessment, Latin America

## Abstract

Nutritional health is essential for older people with dementia. Their feeding is a challenge for which caregivers are not always ready, and an intervention that supports them may have a significant social impact. The aim of this project is to design and evaluate the impact of systematic nursing intervention with formal caregivers to promote nutritional health for older people with dementia. This is a “Nursing Methodology Research” study conducted with formal caregivers of older people with dementia in four Colombian nursing homes. It includes three consecutive phases: (1) systematic intervention design under Whittemore and Grey’s parameters, (2) intervention validation with seven international experts, and (3) measurement of intervention impact, which included a quasi-experimental pre-test–post-test design. The “Nurturing Neurons—Formal Caregivers” intervention met the criteria of systematic health interventions. In response to the work and personal requirements of formal caregivers, the intervention used a tele-support modality. Its content validity ratio (CVR) ranged from 0.88 to 0.92; its content validity index (CVI) was 0.90. The experience was positive for the participant caregivers (94.9%) and professional providers (92.5%). The overall caregivers’ caring competence changed from the medium, 78.1, to the high category, 91.5 (*p* < 0.001). Their perceived burden of care changed from 70.4 to 63.6 (*p* < 0.001). In conclusion, “Nurturing Neurons—Formal Caregivers” achieved a positive impact, with changes in the structure, processes, and outputs to promote the nutritional health of older people with dementia. It led to a significant improvement in formal caregivers’ caring competence and decreased their perceived care burden. Its cost–benefit was favorable; it generated health equity for a vulnerable population and achieved unexpected benefits in the context.

## 1. Introduction

According to the World Health Organization [1], the world is experiencing a significant and accelerated increase in longevity. It estimates that between 2015 and 2050, the share of the world’s population over 60 years of age will increase from 12% to 22%, and that in 2050, 80% of older adults will live in low- and middle-income countries like Colombia.

This increase in longevity poses challenges to social and health systems, which require more resources to meet the demands of this population [2]. As society ages, the support of functionality, autonomy, and well-being in older people becomes an important challenge [3,4]. Longevity brings an increase in the number of people with dementia, which leads to higher pressure on health systems and society. There are currently more than fifty-five million people with dementia in the world, a figure that will reach 152 million in 2050 [5]. In response to this situation, the World Health Organization created a global plan that identified the following necessary actions: giving priority to the issue within public health systems; increasing social awareness and inclusive actions; reducing associated risks; strengthening diagnosis, treatment, and care; supporting caregivers; having information systems; and promoting research and innovation in this field. This plan proposes that 75% of countries should develop policies and programs to support and train caregivers of people with dementia by 2025 [6].

Among the actions related to care for older adults with dementia are food and nutrition. Research indicates that good nutrition is associated with a lower level of cognitive decline and that maintaining adequate nutrition is essential to promote a better quality of life in older adults [7,8]. However, one-third of older adults with dementia have malnutrition, and at least half of them are at risk of developing it, especially in long-term care settings, with malnutrition incidence rates reaching 75.6% and malnutrition risks of 90.4% [9]. Malnutrition in older people with dementia is related to changes in eating behaviors, such as altered hunger signals and difficulty feeding themselves [9]. It is also associated with loss of appetite, decreased olfactory and gustatory perception of flavors, forgetting to eat or drink, difficulties in chewing or swallowing, disruptive behaviors, or mealtime-related events [10]. Poor nutritional status in this group is, in turn, related to greater cognitive and functional deterioration, as well as an increase in neuropsychiatric symptoms, like depression and apathy [11]. There is an urgent need to break this vicious cycle and mitigate the risks of malnutrition in older people with dementia through robust detection and intervention strategies, including dietary modifications and caregiver support measures to improve their nutritional status [12].

A recent systematic search analyzed thirty-three studies on interventions to promote nutrition in older adults with dementia published between 1995 and 2023. Its results report interventions with multiple components that achieved positive effects on food intake: support with meals; environmental modifications that transform the mealtime experience; family-style food offerings; proper presentation of dishes; training for caregivers; and Montessori-type stimulation. According to the researchers, the interventions had a positive effect, with greater knowledge, better attitudes from caregivers, and a meal offering according to the reality and needs of each person and institution. Eighteen studies were conducted in North America, five in Europe, one in Australia, and eight in Asia; none were conducted in Latin America. Most focused on older adults’ nutritional health, with only 21% including formal caregivers. No impact measurement was reported [13].

Caregivers’ and professionals’ views on nutritional health care for people with dementia highlight the importance of accounting for specific conditions, behaviors, and malnutrition risks. They recommend using supplements, environmental management, and addressing caregivers’ stress levels, which can lead to poor food choices, affecting both their health and that of the person they care for [14].

A study on caregivers’ experiences with dementia-related feeding and eating difficulties indicated the impact of dementia on the nutritional status of individuals. Caregivers reported increased levels of stress, which correlated with the level of cognitive impairment of the person they were caring for [15].

Despite the advances, these studies do not address issues like the impact of these situations on families or the stress that families create on care personnel, which can cause greater overload. Moreover, the perception of caregiver burden has a direct impact on the quality of care provided by the caregiver [16]. Therefore, it is important to provide and accept technical training on care, psychoeducation, psychological support, and the development of ethical and emotional tools. This will help bridge the gap between formal caregivers and families, as well as lessen the perceived burden of understanding and accepting this aspect of aging. Supports that enhance caregiving can ease the impact of illness and death and help redefine bereavement and better manage stress [17].

The present study aims to design and rigorously evaluate the impact of a systematic nursing intervention specifically targeted to formal caregivers. The intervention objective is to optimize the nutritional health of older adults with dementia and simultaneously mitigate the burden inherent to the caregiving process. This research seeks to respond to current international commitments that advocate for the well-being of the growing population of older adults with dementia, who are at elevated risk of malnutrition. In addition, it seeks to promote the development and implementation of concrete support strategies for caregivers, enabling them to effectively promote the nutritional health of their patients. Finally, this work seeks to contribute to closing the existing knowledge gap in this area by addressing the impact measurement within the Latin American cultural context, thus enriching the global understanding of this complex study phenomenon.

## 2. Materials and Methods

**Figure 1:**
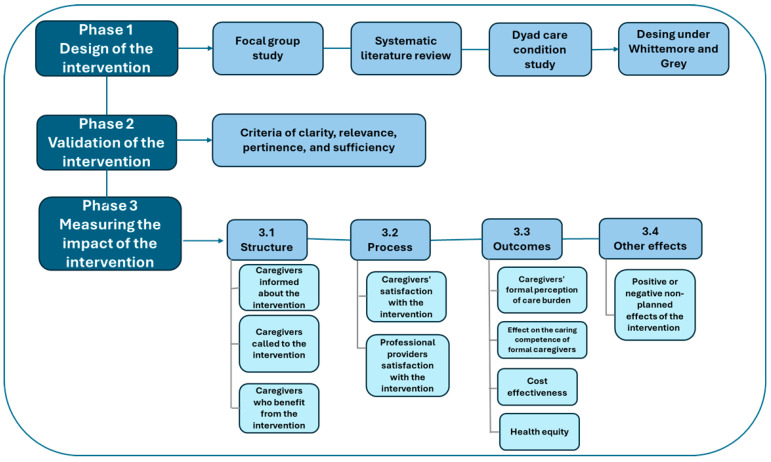
Study phases. Source: Study data, 2025.

This study is a Nursing Methodology Research study. It is part of the project “Nurturing Neurons” 2022–2025. It includes three consecutive phases (see Figure 1).

### 2.1. Phase 1: Intervention Design

The intervention design followed Whittemore and Grey’s guidelines for systematic health interventions. These included finding the problem to be intervened; organizing the content, processes, and conditions for the intervention; and defining its expected effects [18].

Three previous studies from the same research project were inputs for the intervention design: (1) A focus group study with representatives of 14 Colombian gerontological services that described the experience of managers and caregivers concerning feeding and nutrition for older adults with dementia. (2) A systematic literature review that identified the best available evidence on interventions that could improve nutritional nursing care for institutionalized older adults with dementia. (3) An exploratory study that characterized the nutritional care needs of elderly individuals with dementia and their caregivers. Findings from the focus group and characterization studies confirmed the presence of malnutrition or an elevated risk of malnutrition among older adults with dementia. They also reflected a high perceived caregiving burden of formal caregivers, as well as a lack of guidance and skills to address nutritional health problems. Formal caregivers expressed interest in receiving support to develop better caregiving skills, but they perceived a lack of adequate time and space for training in their work environment. However, they indicated frequent use of telephone and computer resources for caregiving support.

### 2.2. Phase 2: Intervention Validation

Seven experts from Latin America validated the intervention. All of them had postgraduate degrees and more than three years of experience with older people with dementia and nutritional health care. They reviewed the intervention’s facial and content validity based on a previously prepared format that rated levels of clarity, relevance, pertinence, and sufficiency. The evaluation format used a Likert scale ranging from one (does not meet the criteria) to four (fully meets the condition evaluated). Each case included space for observations. The results analysis process used Lawshe’s parameters, modified by Tristan, to determine the content validity ratio (CVR) and content validity index (CVI), accepting results above 0.75 as valid [19].

### 2.3. Phase 3: Impact Measurement

We developed the intervention in four elderly care institutions. It included 106 adult formal caregivers who cared for older people with dementia, had access to a mobile device, and had a formal technical or 1.5-year degree.

The intervention impact measurements included its structure, processes, and output. It followed Tanahashi’s framework to enable global understanding [20]. It also included indicators that responded to the five goals proposed by the WHO for the supply of health services: service experience for both receivers and offers, service results, cost effectiveness, and health equity [21,22]. Finally, the impact measurement assessed the intervention’s positive or negative effects according to participants [23] (see Table 1).

Our research group, based on international experiences [24], accepted values of 80% or more as a positive result for the analysis of the structure and process indicators.

We assessed the perception of the care burden for the output indicators based on the section of perception of burden and support of the survey of characterization for the care of the dyad GCPC-UN-D, validated in Colombia [25]. We used this scale because we detected high care burden perception when it was used in the previous characterization study. We considered the survey to be simple, sufficient, and culturally appropriate. The survey scale evaluated the overload perception on a range from zero (none) to three (intense).

Our team used the abbreviated CUIDAR instrument, validated in Colombia, to measure care competence. It assesses knowledge, uniqueness, instrumentation, welfare conditions, anticipation, and support network. Its Cronbach’s alpha was 0.928 [26]. The instrument’s authors state that caring competence is high when scores are above 81, medium when scores are between 47 and 81, and low when scores are below 47.

To evaluate the intervention’s effect, we performed a quasi-experimental pre-test–post-test design. Our participant selection was intentionally from four geriatric homes characterized as part of the previous studies. We evaluated two hypotheses with respect to the effect of the intervention. H_1_0: There is no statistically significant difference in the perception of the burden of formal caregivers before and after the intervention. H_2_0: There is no statistically significant difference in care competence before and after the intervention. Our rejection value for the related samples *t*-test was *p* < 0.05 in both cases.

We avoided study biases of selection, attrition, instrumentation, and measurement. We included participants from institutions with different public and private legal statuses, from different socioeconomic strata, and with both rural and urban locations; we invited all caregivers of the selected institutions to participate in the study. We requested the support of management and institutions to ensure the voluntary participation of formal caregivers in the entire intervention process. We selected standardized and previously defined instruments and measures and asked an external trained professional to collect the measurement data.

To ensure external validity, the results apply only to the Colombian population included but may be potentially beneficial for other groups of a similar nature.

## 3. Results

### 3.1. Intervention Design

The following is a summary of the nursing systematic intervention (see Table 2).

The experts included two nutritionists, four nurses, and a psychologist. Regarding their nationalities, three were Colombians, one was Cuban, one was Chilean, one was Ecuadorian, and one was Venezuelan. The validation results showed a CVR range from 0.88 to 0.98 and a CVI of 0.90 (see the Supplementary Material).

In response to the experts’ suggestions, the researchers included references relevant to the context, activities to strengthen support skills for the caregivers, and clarification of concepts and terms. Our research group also incorporated evaluation details to make it more constructive. Finally, in response to an expert suggestion, the researchers contacted fourteen relatives of these institutionalized elderly patients to inquire about their expectations of care associated with nutritional health and incorporated their suggestions.

### 3.2. Impact Measurement

**Figure 2:**
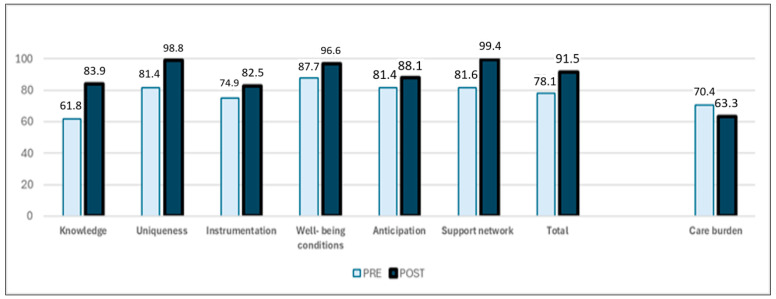
Care competence and perceived burden of formal caregivers. Source: Study data, 2025.

Table 3 and Figure 2 summarize the intervention’s impact (see Table 3 and Figure 2).

### 3.3. Unexpected Effects of the Intervention

This project created a set of interdisciplinary interventions that help individuals and the community. These were the development of innovative programs, the impact on public policies, improvements in quality of life, and the strengthening of academic training and scientific dissemination (see the Supplementary Material).

## 4. Discussion

This study, which measured the impact of the systematic nursing intervention “Nurturing Neurons—Formal Caregivers”, showed positive changes in structure, processes, and products to promote the nutritional health of older adults with dementia. We found positive changes in the structure of the intervention coverage, programmed service coverage, and acceptability of the intervention. Both caregivers and intervention providers reported satisfaction with the process and results. The results showed a positive effect on reducing the perceived burden of care for formal caregivers, effective coverage, improved caregiving competency among caregivers, achievement of cost–benefit ratios with the intervention, and greater health equity.

This systematic nursing intervention is directed at formal caregivers of institutionalized older adults with dementia to promote their nutritional health. Researchers used the results of earlier studies within the same project to leverage the intervention design. This aligns with third-generation universities’ purpose of creating social value based on the development of knowledge [27].

The intervention validation conducted under the Tristan-adjusted Lawshe’s parameters reflected a consensus among the evaluators on the relevance of each of its components [19]. The proposal met the acceptance threshold with expert suggestions. The inclusion of international Spanish-speaking experts helped unify linguistic expressions to make it possible to replicate it in other contexts.

The intervention demonstrated substantial service coverage and received positive acceptance from the participants. The lower-than-expected contact coverage was due to employment status reasons, such as vacation or temporary leave, resignation or retirement, and role changes for the formal caregiver within the institution. It is crucial to evaluate the impact of these modifications on the individuals receiving care and to implement strategies that facilitate reduced employee turnover [28]. It is also necessary to review whether there is an excessive burden of care, which may be associated with caregiver safety, patient behavior, or the time devoted to the people in their care [28].

The intervention aimed to include users and feature measurable, replicable parameters. Both recipients and providers found the intervention inclusive and satisfactory. Dialogue among participants can yield valuable contributions to society [29].

The study results rejected both null hypotheses: there was a statistically significant decrease in the care burden perceived by formal caregivers and a significant increase in their care competence. This achievement in a brief period of six weeks may be explained by the interest and support of the institutions in strengthening the care competency of the caregivers and the backing they received to participate in the intervention.

We designed the intervention in a tele-support modality to conveniently fit formal caregivers’ schedules and locations. It had a total coverage effectiveness.

This type of intervention makes it easier for caregivers to reconcile work, family life, and personal expectations, and we found that it reduced its cost by more than half. Therefore, this project ratifies the recommendation to deliver interventions in diverse ways to respond to the reality of caregivers of people with dementia [30]. This type of intervention has the potential to help caregivers reduce the malnutrition risk in elderly people with dementia and avoid other complications, including an accelerated cognitive decline [31]. However, we need to continue this research to demonstrate if it helps in such topics.

Our intervention modality shows similar effects to the intervention developed with a telehealth coaching modality, which also found positive effects on caregivers of people with dementia nutrition [32].

The reported interventions to improve the feeding and nutrition of older adults with dementia aimed to improve their participation, compensate for their dysfunction, and increase food intake through nutritional supplementation, assistance, person-centered care, environmental modifications, education, and other multicomponent actions [21]. This study supports the importance of person-centered care and assessments to train and motivate staff to provide this care [33,34]. Our research evaluated the nursing systematic intervention impact, unlike previous studies.

Our findings support previous researchers’ findings that identified ways to optimize formal caregivers’ ability to feed individuals with dementia. They reported that the processes associated with nutrition are complex and that caregiver care competence plays a key role in positively impacting the eating experience of individuals with dementia [35,36]. We also agree with a study on caregivers who experience challenges related to the nutritional health of older adults with dementia in their care. The results emphasize the importance of receiving information and support regarding nutritional care from health professionals to develop management strategies and to reduce the burden of caregiving [37].

This systematic nursing intervention strengthens the knowledge, attitude, and behavior of formal caregivers of people with dementia at mealtimes. These may include, as reported in previous studies, taking care of their posture and comfort; verbal and nonverbal communication; their physical orientation; food description and respect for preferences; portioning; the variety of foods and beverages offered; ensured schedules; cleanliness of the person and their environment; and manifestations of commitment and closeness to them [35].

This project responds to the needs of caregivers of older people with dementia. It is necessary to consider their own opinion regarding the training they have or require. While it is important to provide them with increased caregiving support, it is also important to ensure that they have the knowledge and skills needed to perform their role [38]. Additionally, the researcher’s selection of the institutions taking part in this research reflected equity in the proposed supply of health services [21].

Our group responded to global demand in the field of nutritional health of people with dementia. Our results have a positive impact on the structure, process, and expected products. In this sense, this field of study requires continued expansion to broaden the scope of this systematic nursing intervention. This impact evaluation study included participants as a fundamental principle to ensure effectiveness and to promote equity and sustainability [23,29]. Despite these findings, this study has the limitation of not having a randomized controlled experimental design that would improve the level of evidence of these results.

## 5. Conclusions

The nursing systematic and validated tele-support intervention “Nurturing Neurons—Formal Caregivers”, which promotes nutrition in older people with dementia, achieved a positive impact, with changes in the structure, processes, and outputs that promote the nutritional health of older people with dementia.

Its design responded to formal caregivers’ needs and contexts. It reflected good overall coverage and programmed care and was well accepted, as evidenced by its completion by caregivers who kept their work positions.

The intervention led to elevated levels of satisfaction among recipients and providers. It significantly decreased the perceived burden of care for formal caregivers and increased their caring competence, while having effective coverage, a net benefit, and greater health equity for the vulnerable population.

The impact evaluation design revealed unexpected results that positively affected the community involved. This input can be the basis for public policies that respond to international commitments for the care of this population group.

Given the positive impact of this intervention, it will be relevant to advance this research field by strengthening the emotional, bioethical, and educational aspects and involving both formal caregivers and family members to strengthen the nutritional health care of older people with dementia while reducing the care burden for their caregivers.

## Figures and Tables

**Table 1:** Impact measurement.

Type of Indicators	Purpose of Measurement	Applied Formula
Structure	Intervention coverage	[(Formal caregivers called/Formal caregivers needing the intervention) × 100]
	Programmed service coverage	[(# of formal caregivers registered/Formal caregivers summoned to take part in the intervention) × 100]
	Acceptance of the intervention	[(# of Formal caregivers who attended the intervention/# of Formal caregivers registered in the intervention) × 100]
	Contact coverage	[(# of Formal caregivers who completed the intervention/# of Formal caregivers registered) × 100]
Process	Satisfaction of formal caregivers with the intervention	Satisfaction perception % with the intervention’s novelty, usefulness, importance, approach to the topic, and overall experience, obtained in a survey
	Satisfaction of professional providers with the intervention	Satisfaction perception % with the intervention’s novelty, usefulness, importance, approach to the topic, and overall experience, obtained in a survey
Output	Effect on the formal caregiver’s perception of care burden	Difference in the means between the level of perception of burden of care before and after the intervention
	Coverage effectiveness	The % of formal caregivers who received help from the program: [(# of formal caregivers who improved their caring competence after the intervention/# of formal caregivers who completed the intervention) × 100]
	Effect on the caring competence of formal caregivers	Difference in the means between the level of caring competence of formal caregivers, globally and by components, before and after the intervention
	Cost–benefit of the intervention	[Cost in US$ calculated by the institution for the conventional intervention-cost in US$ of the flexible intervention]
	Health equity	Detail verification of the inclusion of public and private institutions, with different socioeconomic and geographical conditions

Source: study data, 2025.

**Table 2:** Summary of the nursing systematic intervention.

*Intervention Section*		*Section Description*	
*General description*	Name	Nurturing Neurons—Formal Caregivers	
	Issue to resolve	Insufficient care competence in formal caregivers of elderly people with dementia, at risk of nutritional health impairment.	
	Addressed recipients	This intervention targets formal caregivers addressing the nutritional needs of institutionalized individuals aged 60+ with severe neurocognitive disorders that impact daily activities.	
	Modifiable aspects through the intervention	The modifiable aspects are those related to the improvement in the formal caregivers’ care competence related to nutrition and feeding of the cognitively impaired elderly in their care. These include each of the domains that make up the competence: the level of knowledge; the recognition of the unique conditions as a caregiver; how to make the therapeutic instructions on nutrition and feeding of the elderly operational; the identification and management of the welfare conditions available to support feeding and nutrition; the ability to anticipate and minimize risks associated with feeding and nutrition; and the identification and consolidation of a support network to care for aspects related to feeding and nutrition.	
	Appropriate conditions	The conditions for the development of the intervention include generating an environment of trust, presenting the content of the educational material and explaining it before using it, conducting feedback and re-teaching processes with the participants, and explicitly thanking them in each session for their attention and dedicated time.	
	Necessary resources	Professional staff with experience in the work of nutritional care of the elderly with cognitive impairment and their formal caregivers. Materials for education, ability reinforcement, and field exercises in various presentation modalities. Connectivity and availability of materials to achieve access through a mobile phone.	
*Intervention development*	Processes for its development	The intervention uses tele-support. It includes six sessions in a total of fourteen hours, as follows: two hours for each of the six dimensions addressed (one hour of training and one hour of independent work), plus two hours for the induction and evaluation. The frequency of meetings is biweekly.	
	Expected results	Proximal	Level of perception of satisfaction of the formal caregiver. Level of knowledge, uniqueness, instrumentation, provision of well-being conditions, anticipation, and ability to recognize and generate a support network to address feeding and nutrition.
		Primary	Level of caring competence of the formal caregiver to address feeding and nutrition.
		Secondary	Feeding practices and levels of nutrition.
		Distal	The design of a menu that considers different textures and ensures an adequate nutritional balance. Guidelines that serve as a basis for a public policy about the care of people with cognitive impairment and support for their formal caregivers.
*Contents* *of the intervention*	Phase 1: “Who we are”	Session 1	Introduction to the intervention. Presentation of the participants to learn about their expectations and for them to propose joint goals for nutritional care for the older adults in their care. Presentation of the generalities of the intervention. Addresses the topics of uniqueness and provision of well-being conditions.
		Session 2	Reinforcement of the topics of uniqueness and provision of well-being conditions through individual and group independent work exercises.
	Phase 2: “How we care”	Session 3	Addresses the topics of knowledge and instrumentation.
		Session 4	Reinforcement of the topics of knowledge and instrumentation through individual and group independent work exercises.
	Phase 3: “Who we count on”	Session 5	Addresses the topics of anticipation and support network.
		Session 6	Reinforcement of the topics of anticipation and support network through individual and group independent work exercises. Final evaluation of the intervention, checking whether it met the established expectations and goals.

Source: Study data based on Whittemore and Grey’s parameters, 2025.

**Table 3:** Impact results of the intervention.

Type of Indicator	Name	Results	
Structure	Intervention coverage	100%	
	Programmed service coverage	86.5%	
	Acceptability of the intervention	86.8%	
	Contact coverage	70.7%	
Process	Satisfaction with the intervention	Formal caregivers	94.9%
		Professional providers	92.5%
Output	Effect on the formal caregiver’s perception of care burden	Burden of care perception diminished in 7.1% (*p* < 0.001)	
	Coverage effectiveness	100%	
	Effect on the care competence of formal caregivers	Global care competence—13.4% changing from the medium to high level (*p* < 0.001)	
	Cost–benefit of the intervention	Prior achievement of the purpose: Cost of the conventional modality: USD 36,099.91 Cost of the tele-support modality: USD 15,354.18 Savings: USD 20,276.90 (−56.2%)	
	Health equity	Participant institutions included different geographical areas, were both public and private, and had different socioeconomic levels	

Source: Study data, 2025.

## Data Availability

The data are available on request from the corresponding author. The authors are interested in contacting different organizations that are interested in the same topics.

## Supplementary Materials

The following supporting information can be downloaded at https://www.mdpi.com/article/10.3390/ijerph22060849/s1: Supplementary material Table S1: Intervention validation results; Supplementary material Table S2: Unexpected effects of the intervention.
