# Supplementary material for: Systematic Intervention with Formal Caregivers to Promote Nutritional Health of Older People with Dementia: An Impact Evaluation Study
Source: Int J Environ Res Public Health. 2025 May 29;22(6):849. doi: 10.3390/ijerph22060849 (PMC12192597; doi:10.3390/ijerph22060849)
Supplement: Supplementary file 1 [file ijerph-22-00849-s001.zip › ijerph-3598124-supplementary.pdf]

Supplementary material Table S1. Intervention validation results

DIMENSION	COMPONENT	CLARITY %	COHERENCE %	RELEVANCE %	SUFICIENCY %	CVR
**General description**	Problem to be addressed	89,3	92,9	92,9	78,6	0,88
	Subject to whom it is addressed	85,7	92,9	92,9	78,6	0,88
	Intervention route	96,4	92,9	96,4	78,6	0,91
**Intervention development**	Development format	92,9	96,4	100	82,1	0,93
	Expected results	92,9	92,9	96,4	82,1	0,91
**Intervention contents**	Session 1	85,7	96,4	92,9	82,1	0,98
	Session 2	89,3	92,9	92,9	82,1	0,89
	Session 3	92,9	96,4	92,9	78,6	0,91
	Session 4	92,9	96,4	92,9	78,6	0,9
	Session 5	92,9	96,4	96,4	78,6	0,91
	Session 6	92,9	96,4	96,4	82,1	0,92
					**CVI**	**0,9**

Source: Study data, 2025.

Supplementary material Table S2. Unexpected effects of the intervention

An ecological and decorative garden "Cultivating Smiles", design by students together with older adults in the gerontological services. This was carried under a university pilot program of interdisciplinary experiential learning.
An implementation of a reality-oriented scheme with items that helped older people to frame their time and space perception. It included a support of books specially selected for reminder workshops and donated by the university library.
The approval of a national law project for the protection of family caregivers of dependent persons in which the researcher group participated.
Development and delivery of specific menus for elderly people with dementia based on Colombian gastronomy with the participation of food engineers and gastronomes and the validation of nutritionists.
Review of the quality of the culinary processes of participating institutions that helped in their quality improvement.
Decrease of 71% in the amount of food wasted according to the official report that was monitored in one of the institutions during the time the research was conducted.
Development of master's degree in nursing thesis on the effect of doll therapy in people with dementia.
A doctoral thesis in nursing with a randomized controlled trial for the evaluation of the effect of a systematic nursing intervention to support the role performance of family caregivers of people with dementia.
The involvement of ten undergraduate students with products that include: two booklets, one to guide the nutrition of the elderly and another to promote reminiscence therapy based on nutrition.
The support for the systematization of institutional clinical records of six systems engineering students.
The scientific repercussion was identified as a relevant factor with two publications in high impact journals, as well as the permanent dissemination of these results in papers and press releases and the invitation to replicate them to fifty-six gerontological institutions in Colombia.

Source: Study data,2025
